# Combination of Noble Metal and Gold–Silver Nanoclusters as Enhanced Antibacterial Coatings for Ti-Based Medical Implants

**DOI:** 10.3390/ijms262411945

**Published:** 2025-12-11

**Authors:** Evgeniia S. Vikulova, Svetlana I. Dorovskikh, David S. Sergeevichev, Tatiana Ya. Guselnikova, Anastasiya D. Fedorenko, Alexander A. Zheravin, Natalya B. Morozova

**Affiliations:** 1 Nikolaev Institute of Inorganic Chemistry, Siberian Branch of the Russian Academy of Sciences, 3 Lavrentiev Ave., 630090 Novosibirsk, Russia; reter16@yandex.ru (S.I.D.); tguselnikova@niic.nsc.ru (T.Y.G.); fedorenko@niic.nsc.ru (A.D.F.); mor@niic.nsc.ru (N.B.M.); 2 E. Meshalkin National Medical Research Center, Ministry of Health of the Russian Federation, 15 Rechkunovskaya Str., 630055 Novosibirsk, Russia; d_sergeevichev@meshalkin.ru (D.S.S.); zheravin@meshalkin.ru (A.A.Z.)

**Keywords:** noble metals, titanium alloy implants, film heterostructures, AgAu nanoclusters, chemical vapor deposition, physical vapor deposition, antibacterial activity, histological study

## Abstract

The surface modification of medical implant materials stands as a favorable strategy to enhance their biological properties including their antibacterial effect and biocompatibility. Recently, both in vitro and in vivo studies have shown that film heterostructures based on a combination of noble metal sublayers and an active component, such as silver and gold nanoparticles, offer unique advantages. The present work develops this promising direction and focuses on a series of combinations of noble metal coatings functionalized with bimetallic nanoclusters obtained by vapor-phase deposition methods onto the surfaces of Ti-based implants. This investigation investigates the influence of sequential deposition (AgAu or AuAg) and noble metal component (Ir or Au) on the coating morphology and the active component chemical form and release. Thus, scanning electron microscopy, X-ray photoelectron spectroscopy, and inductively coupled plasma atomic emission spectroscopy have been applied to characterize the samples before and after in vivo biological studies (rat models, 1 and 3 months). Histological and blood analyses confirmed the high biocompatibility of all the heterostructures. The samples also showed a pronounced in vitro biocidal effect against Gram-positive (*S. epidermalis*) and Gram-negative (*P. aeruginosa*) bacteria that correlates with a dynamic of silver release. The AuAg/M heterostructures demonstrated superior biological characteristics compared to their AgAu/M counterparts, suggesting enhanced both long-term integration and antibacterial action.

## 1. Introduction

Titanium and its alloys form the bedrock of modern implantology, with their applications spanning dentistry, orthopedics, cardiac, and oncological surgery [1,2,3]. Known for their biocompatibility [4,5], high strength, elasticity [2], resistance to corrosion, and a long service life [4,6], titanium-based materials, such as commercially pure titanium and its alloyed counterpart [7,8], are often used in the creation of diverse implants. Recent innovation has turned toward 3D, mesh structures [9,10]—a “titanium silk” that promises enhanced performance. These mesh implants possess increased strength [11,12] and multi-directional extensibility, perfectly conforming to the anatomical area of implantation. The enhanced structure of meshes ensures their improved adhesion to surrounding tissues. Clinical tests offer evidence: titanium mesh has reduced inflammatory responses in the treatment of postoperative ventral hernias [13].

However, the specter of peri-implant infections remains a key problem, impairing the life quality of post-implantation patients [14]. This problem is amplified by the ability of bacteria to form biofilms and develop resistance to a growing amount of antibiotics [15,16]. There are two main strategies for addressing this problem [17]. In particular, new titanium-based alloy compositions incorporating antibacterial components, such as Ti-Cu and Ti6Al4V-Cu, are being actively developed [18,19]. This is an attractive approach, but it results in a sudden release of active component ions [20], which minimizes the antibacterial effect of the implant in the long term postoperative period.

A more versatile alternative is the application of specialized coatings that could provide time-tunable antibacterial action. This can be achieved through a combination of active agents and/or interaction between the surface and the antibacterial component. For this purpose, film (hetero)structures based on a combination of inert noble metals (Ir, Au, and Pt) with an active component (Ag, Au, and Cu) are highly promising [21,22,23]. In this case, a galvanic (Ag, Cu) or synergistic effect (Au) is expected, involving controlled release of the active component ions in order to remain it within safe therapeutic limits [24,25,26]. The next step to provide the fine tuning of the dynamics of antibacterial activity and biocompatibility is the application of silver-based nanoalloys as antibacterial components [27,28,29,30]. Moreover, the combination of Ag and Au allows for the mitigation of the disadvantages of both metals, such as undesirable reactions in the body (silver) or insufficient antibacterial action (gold). In vivo tests showed that AgAu nanoalloys were nearly 11,000 times more effective than gentamicin in healing skin wounds in mice infected with MDR MRSA [31]. AgAu nanoparticles promoted faster healing and regeneration of infected wounds without scar formation [32].

**Figure 1:**
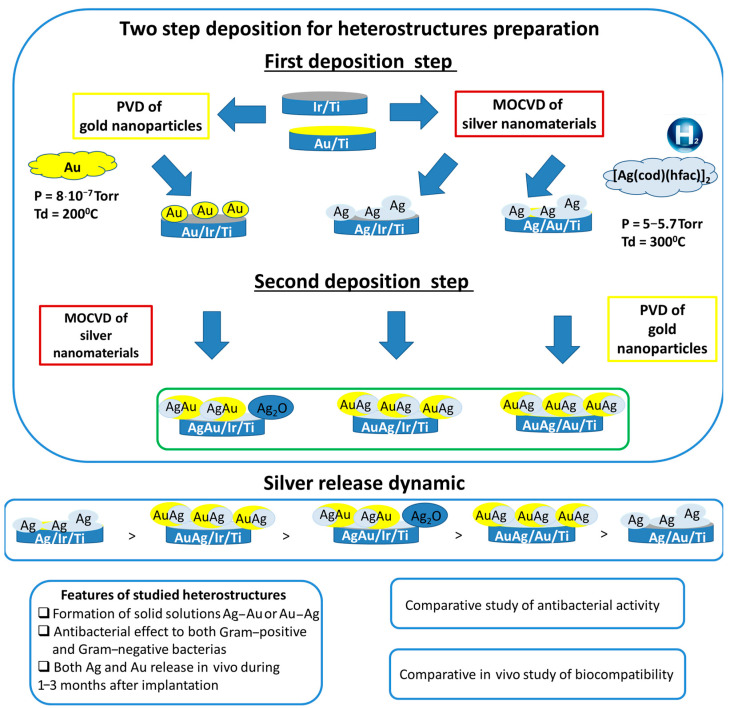
The general summary of sample preparation and testing.

Thus, the current work is devoted to the production and primary biological characterization of the new types of heterostructures consisting of an iridium or gold sublayer decorating with bimetallic gold–silver nanoclusters (Figure 1). Note that AgAu nanoparticles are typically obtained by solution deposition methods [33], electrochemical deposition [34,35,36], and green chemistry methods [27,28]. Along with traditional approaches, gas-phase methods (physical vapor deposition (PVD) and metalorganic chemical vapor deposition (MOCVD)) are highly effective, providing controlled growth of coatings and nanoclusters with a specific composition, microstructure, and morphology on complex-shaped products [24,25,37], which is relevant in the case of 3D mesh implants.

In this work, the gas-phase methods were adopted for the surface modification of Ti-based implants. The target nanoalloy composition was enriched in silver, as it is the more biologically active component (Ag/Au component ratio close to two) [38]. The influence of the deposition sequence of the bimetallic components was investigated (Figure 1). The composition, microstructure, release dynamics of active components, and biocidal action of the heterostructures were first studied. In vivo blood composition studies were conducted, and histological parameters were assessed after implantation in experimental animals (rats).

## 2. Results

### 2.1. Samples Characterization

The process for preparing a series of samples for the study is described in detail in Section 4.1. Initially, the continuous iridium and gold sublayers were deposited onto titanium supports by a MOCVD method (Figure S1). The binding force of Ir and Au coatings with Ti disks were estimated visuality using tape with 10 N/cm. For both samples there were no signs of noble metal coatings on the tapes. This adhesion characteristics are comparable to those described for metal layers [39] and hydroxyapatite coatings [40].

Next, the two-step deposition process was performed, proposed to obtain bimetallic nanoclusters on bare and coated substates (Ti, Ir/Ti, and Au/Ti, Figure 1). The designations “AgAu” or “AuAg” were used to indicate the order of metal deposition. In particular, the deposition Au nanoparticles, first, and then Ag nanomaterials onto Ti and Ir/Ti, second, gave the samples AgAu/Ti and AgAu/Ir/Ti, respectively (Figure 1) The reverse deposition sequence (Ag first, Au second) was employed for the preparation of AuAg/Ti, AuAg/Ir/Ti, and AuAg/Au/Ti heterostructures (Figure 1). According to ICP-OES data, the Ag/Au ratio in all the obtained samples lied in the range of 1.8–2.3. The effect of the deposition sequence on the morphology and chemical form of the active component is clearly evident, depending on the substrate surface.

**Figure 2:**
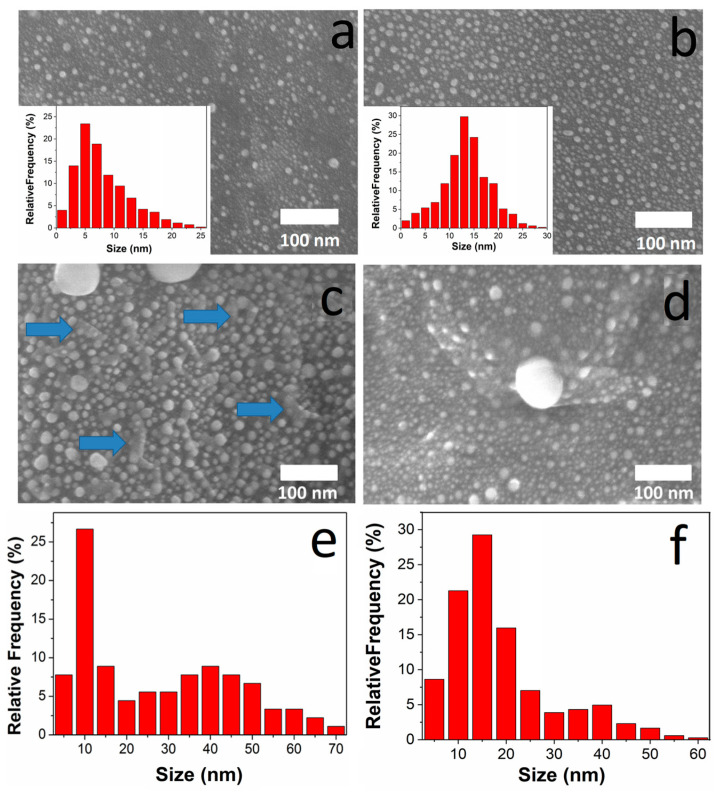
Typical SEM images with size distribution for Au/Ti (**a**), Ag/Ti (**b**), AgAu/Ti (**c**,**e**), and AuAg/Ti (**d**,**f**) samples (the blue arrows pointed on the elongated structural elements).

For bare Ti substrates, the main difference when changing the Ag and Au deposition sequence is observed in the particle sizes and their distribution patterns (Figure 2). Prior to the second step deposition, only Au or Ag spherical nanoparticles lower than 30 nm exist on the Ti surface (Figure 2a,b). After the application of the second active component, spherical particles are also formed (Figure 2c,d), but with a bimodal size distribution (Figure 2e,f). Moreover, comparison of both pairs of the samples shows a noticeable increase in the number of larger particles and a shift in the size maxima to a larger region (from Ag to AuAg: from 12 to 17 nm; from Au to AgAu: from 5 to 10 nm). This may indicate the formation of the bimetallic nanoclusters. Furthermore, for the AgAu/Ti sample, novel elongated structural elements are also observed (Figure 1, marked with the blue arrows), probably arising from interaction between the Ag and Au components.

**Figure 3:**
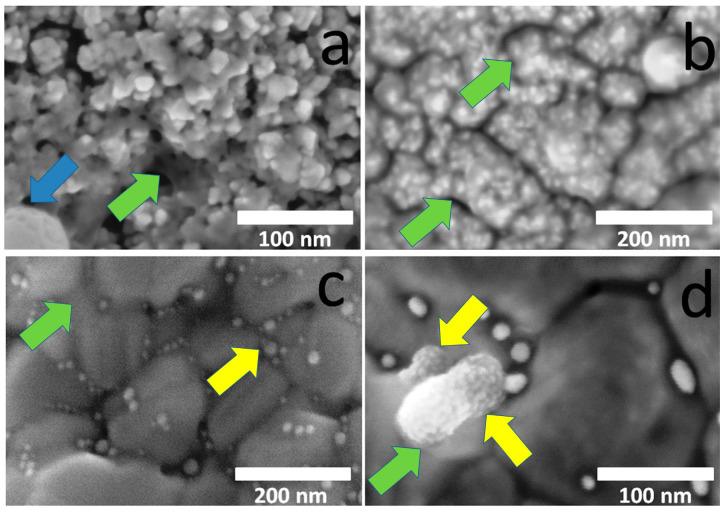
SEM images of surfaces of AgAu/Ir/Ti (**a**), AuAg/Ir/Ti (**b**), and AuAg/Au/Ti (**c**,**d**) samples (the blue arrow pointed on individual grains belonged to Ag_2_O, the green arrows pointed on thin Ag film, and the yellow arrows pointed on individual grains belonged to AuAg).

For noble metal-coated Ti substrates, other features of the active component formation are observed. In fact, the gold is formed as small nanoparticles while the silver is mostly formed as thin films (Figure 3, marked with the green arrows), enveloping both the crystallites of the Ir or Au sublayer (AuAg/Ir/Ti and AuAg/Au/Ti samples) or the Au nanoparticles (AuAg/Au/Ti samples). This is consistent with previous studies for monometallic Ag or Au active components [24,25]. However, after the two-stage deposition of the active component, novel types of particles are also observed, namely, enlarged individual grains of 20–100 nm (Figure 3, marked with blue and yellow arrows). Analysis of the chemical composition (see below) allows us to classify them as Ag_2_O (AgAu/Ir/Ti sample, blue arrow, Figure 3a) and AuAg (AuAg/Au/Ti sample, yellow arrows, Figure 3d), respectively. Thus, during the deposition of silver in the case of the AgAu/Ir/Ti sample, part of the silver surface does not contact with gold and the Ir sublayer and is actively oxidized, which distinguishes this sample from its analogs (AuAg/Ir/Ti and AuAg/Au/Ti samples). This is apparently due to the fact that silver, aggregating as individual nanoclusters rather than as a thin film, no longer has contact with gold nanoparticles, preventing the charge transfer that stabilizes it from oxidation [41].

To better ascertain the impact of the Au component on the morphological features of heterostructures, the comparative analysis of the AgAu/Ir/Ti sample with the previously obtained Ag/Ir/Ti one [24] has been performed using SEM-AFM (see details in Supplementary Information). For the AgAu/Ir/Ti sample, the half width of a single grain is 49 nm while the grain height is 19 nm (Supplementary Information, Figure S2a). The grain diameter distribution statistics (Supplementary Information, Figure S2b,c) indicate the formation of grains with diameters from 16 to 130 nm with a median value of ~62 nm. Without the Au component (sample Ag/Ir/Ti [24]), the half width of a single Ag grain is 48 nm while the grain height is 12 nm (Supplementary Information, Figure S2d). This is comparable to the AgAu/Ir/Ti sample. However, Ag/Ir/Ti is characterized by a wide particle size distribution with a median value of ~80 nm (Figure S2e,f). The analysis of the surface topology showed that the modification of Ir/Ti with bimetallic particles leads to an increase in the roughness of the samples to 79 nm (AgAu/Ir/Ti), which is higher than the roughness values for heterostructure samples modified only with Ag particles [24].

**Figure 4:**
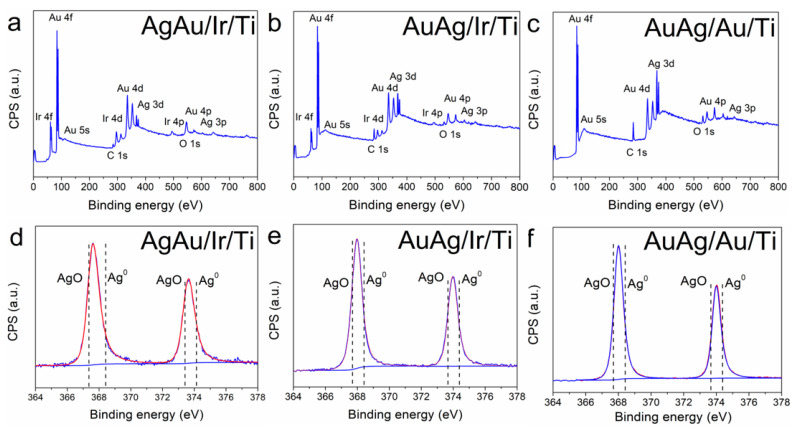
Survey XPS spectra and Ag 3d spectra of samples: AgAu/Ir/Ti (**a**,**d**), AuAg/Ir/Ti (**b**,**e**), and AuAg/Au/Ti (**c**,**f**) samples.

The composition of AgAu/Ir/Ti, AuAg/Ir/Ti, and AuAg/Au/Ti samples was studied using XPS (Figure 4a–c). In all the samples, only one chemical state of Ag was observed (Figure 4d–f) that could be assigned as intermediate between the metal (Ag^0^, 3d_5/2_ = 367.8 eV [42]) and the oxide (Ag^+^-O, 3d_5/2_ = 368.3 eV) [43]. The positions of Ag 3d_5/2_ peaks for the AuAg/Ir/Ti and AuAg/Au/Ti samples are close (368.0 eV, Figure 4e,f), while this position was slightly shifted to the oxide region for the AgAu/Ir/Ti sample (368.1 eV, Figure 4d). This intermediate state of silver was not detected for previously studied Ag/Ir and Ag/Au heterostructures with a monometallic active component [24,25]. These observations indicate a pronounced interaction between Ag and Au particles, which is characteristic of nanoalloys [44]. The gold in all the samples is present in the metallic state (Au^0^ 4f_7/2_ = 83.9–84 eV) [45] (Figure S3a–c). After etching the AuAg/Ir/Ti and AuAg/Au/Ti samples to a depth of 2–3 nm, the oxygen content almost disappeared (less than 1.5 at.%), indicating the absence of a Ag oxide phase in the surface layers. Upon deconvolution of the O1s spectra of the AuAg/Ir/Ti and AuAg/Au/Ti samples (Figure S3d–f), the two oxygen states belonging to the C=O group (at 531.6–532 eV) and belonging to the O-H group (at 534–534.2 eV) [46] were revealed. For the AgAu/Ir/Ti sample, the total oxygen content in the surface layer after etching was 4.9 at.%, while the Ag content was 4.8 at%. According to O1s spectra, the additional oxygen state with the position at 530.9 eV [47] also appeared (2.1 at.%). This pointed to the presence of a silver oxide phase in addition to the intermediate state of Ag (Figure 4d). Since the Ag/O ratio was estimated to be two, this oxide phase seems to be an Ag_2_O.

Thus, the sequence of the deposition of the bimetallic active component affects the form of the active component deposited on the surface of the noble metal.

In the case of the AuAg/Ir or AuAg/Au heterostructures, silver is first deposited on the surface of the Ir and Au coatings, forming a nanofilm and individual clusters. Subsequently, gold deposition occurs, resulting in the formation of Ag-Au solid solutions on the surface, as a result of gold–silver interaction.

In the case of AgAu/Ir, gold is first deposited on the surface of the Ir coating, forming individual gold nanoparticles rather than a film, resulting in partial blocking of the iridium surface. In the second stage, silver is deposited on the surface, forming a silver nanofilm on the surface of the gold particles and the Ir coating (this nanofilm interacts with gold to form a bimetallic phase) and individual silver clusters (which oxidize in the absence of contact with gold).

### 2.2. Dynamics of Metal Ions’ Release

The dynamics of metal ion release from the surface of the samples were studied in the model solution of sodium phosphate buffer (pH = 6.8).

**Figure 5:**
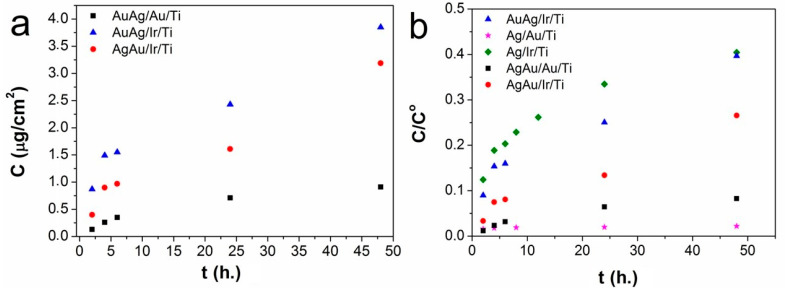
Study of the dynamics of silver ion release from the surface of heterostructure samples in the coordinates C_Ag+_—t (C_Ag+_ concentration of silver released into the solution, μg/cm^2^ from t time in hours) (**a**), and the proportion of Ag^+^ C_Ag+_/C_0_—t (C_Ag+_/C_0_ concentration of silver released into the solution normalized to the initial concentration from t time in hours) (**b**).

For the silver dissolution, the results are presented in Figure 5a, as dependencies of *C* (metal concentration in solution, μg/cm^2^) versus *t* (time, hours). For all samples, an increase in the dynamics of silver release is observed in the starting hours and slows down after 24 h. It is shown that this process is most influenced by the sublayer metal. Thus, the lowest release rates were observed in the case of AuAg/Au/Ti (0.02–0.06 μg/cm^2^h), while for AuAg/Ir/Ti (0.08–0.6 μg/cm^2^h) the release rate in the starting hours was almost an order of magnitude higher. For AgAu/Ir/Ti, the release rates (0.06–0.2 μg/cm^2^h) were lower than for AuAg/Ir/Ti. Thus, the presence of an iridium sublayer and direct contact of the Ag/Ir galvanic couple accelerates this process.

To evaluate the role of bimetallic nanoalloys on the dynamics of silver release from the surface of heterostructures, the comparison of the data on silver ion release for the AgAu/Ir/Ti, AuAg/Ir/Ti, and AuAg/Au/Ti samples with data for the Ag/Ir/Ti and Ag/Au/Ti samples with similar silver contents, obtained by us earlier [24], was carried out. For this purpose, more versatile dependencies were constructed (Figure 5b): fraction of silver—*C*/*C*^0^ (concentration of silver released into the solution/initial concentration of silver) versus *t* (time, hours). It was shown that the fraction of Ag^+^ ions released from the surface during the first few hours is at its maximum (0.2) for the Ir sublayer (Ag/Ir/Ti) and minimum (0.01) for the Au sublayer (AuAg/Au/Ti and Ag/Au/Ti) (Figure 5b). At 48 h, the fraction of Ag^+^ ions changes in the following series: Ag/Ir/Ti ≈ AuAg/Ir/Ti (0.4) > AgAu/Ir/Ti (0.26) > AuAg/Au/Ti (0.08) > Ag/Au/Ti (0.02), and is at its maximum for the Ag/Ir/Ti and AuAg/Ir/Ti samples with direct Ag/Ir contact. Thus, the previously studied Ag/Ir/Ti and Ag/Au/Ti samples are “boundary” structures in this series. For the AuAg/Au/Ti and Ag/Au/Ti samples with direct Ag/Au contact, the proportion of Ag^+^ ions differs by a factor of four. The presence of small Au particles likely increases the surface area of the AuAg/Au/Ti sample, stimulating a more active release of ions from its surface compared to that from the Ag/Au/Ti sample. Another reason for the slow dissolution of silver ions from the Ag/Au/Ti surface is the presence of an oxide phase.

In contrast with the silver component, the release of Au^+^ ions was not detected in the period of 2–48 h, within the sensitivity limits of ICP-OES method. Thus, for all samples, the final concentration of gold into the solution after 48 h did not exceed 3 × 10^−2^ μg/cm^2^.

### 2.3. Antibacterial Study of Heterostructures with Bimetallic Nanocomposites

**Figure 6:**
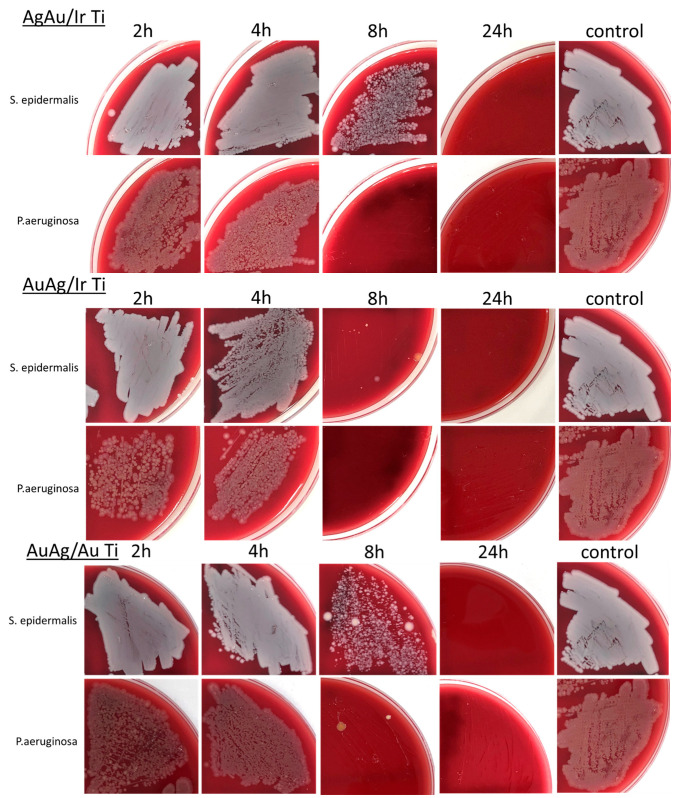
Reduction in the number of *S. epidermalis* and *P. aeruginosa* colonies with increasing incubation duration on the surface of samples with different variants of heterostructure coatings.

**Figure 7:**
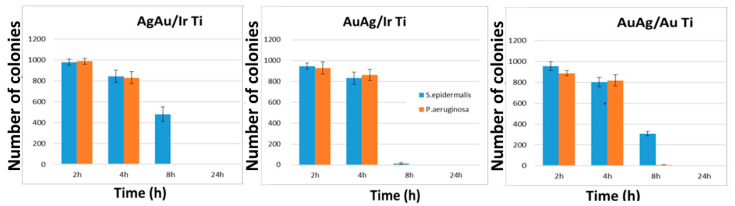
Decrease in colony growth dynamics after incubation on the surface of AgAu/Ir/Ti, AuAg/Ir/Ti, and AuAg/Au/Ti samples for 2, 4, 6, 24, and 48 h.

All tested samples of AgAu/Ir/Ti, AuAg/Ir/Ti, and AuAg/Au/Ti were active against *P. aeruginosa* and *S. epidermalis* strains (Figure 6). The antibacterial effect increased with increasing incubation time. Quantitative data are presented in Table S2. After 24 h, no viable microorganisms capable of forming colonies and growing were detected. A significant difference was recorded for Gram-positive (*S. epidermalis*) and Gram-negative (*P. aeruginosa*) bacterial strains. In all cases, 8 h of incubation on the sample surface was sufficient to suppress the growth of *P. aeruginosa* (Figure 6 and Figure 7). However, a comparison of AgAu/Ir/Ti and AuAg/Ir/Ti with their previously studied analog Ag/Ir/Ti [23] showed that the bactericidal effect of bimetallic nanoalloys was less pronounced: for Ag/Ir/Ti, inhibition of *P. aeruginosa* bacterial growth occured after 2 h. Interestingly, in the case of the AuAg/Au/Ti sample, the inhibition of *P. aeruginosa* growth also occured by 8 h, despite its lower share of silver ion release compared to the AgAu/Ir/Ti, AuAg/Ir/Ti, and Ag/Ir/Ti samples. Apparently, even a low content of Ag^+^ ions is sufficient to inhibit the growth of Gram-negative bacteria. For Gram-positive *S. epidermalis* bacteria, the antibacterial effect of the heterostructures is less pronounced. For AuAg/Ir/Ti, which provides the most effective Ag dissolution due to the galvanic effect, inhibition is almost complete (<2% surviving bacteria). This is consistent with the relative dynamics of silver dissolution (Figure 5b). In contrast, samples with lower dissolution dynamics inhibit *S. epidermalis* growth only after 8 h.

### 2.4. In Vivo Biocompatibility Studies

**Figure 8:**
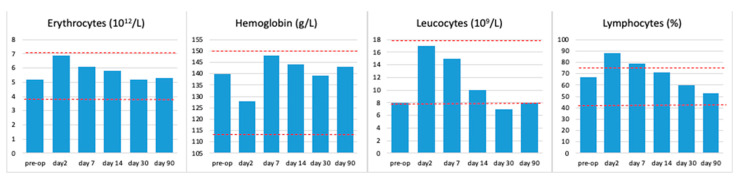
Dynamics of general blood test parameters at different observation periods. The upper and lower limits of the physiological norm are marked with a red dotted line.

**Figure 9:**
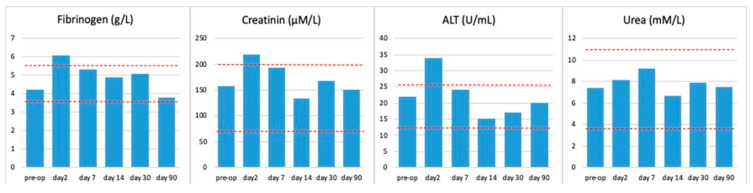
Dynamics of biochemical blood parameters at different observation periods. The upper and lower limits of the physiological norm are marked with a red dotted line.

**Figure 10:**
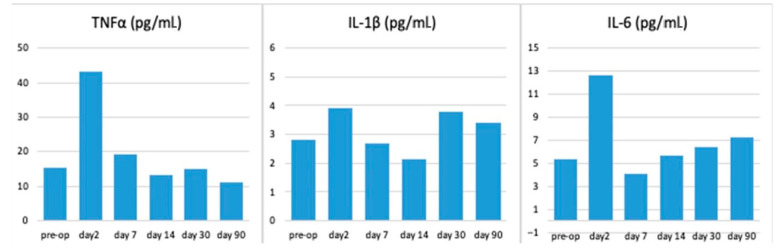
Dynamics of the level of proinflammatory cytokines in rat blood serum at different observation periods.

For the first time, the dynamics of the cytological, biochemical, and cytokine composition of peripheral blood in laboratory rats, at various implantation times, for samples with AgAu nanocomposites was studied. A complete blood count was performed, which included the determination of hemoglobin, red blood cell count, white blood cell count, and lymphocyte percentage. Furthermore, biochemical blood tests were conducted to determine the levels of fibrinogen, creatinine, alaninaminoransferase, and urea. In addition to these conventional analyses, immunological blood tests were performed to ascertain the serum concentrations of TNF-α, IL-1β, and IL-6. Data from animals in all groups were collected and averaged for each parameter studied at different time periods (Figure 8, Figure 9 and Figure 10). The results of the complete blood count indicated that the parameters under investigation remained within the standard physiological limits (see Figure 8). An increase in the percentage of lymphocytes was noted on days two and seven after surgery. By the second week of observation, this parameter had returned to preoperative values.

The study of blood biochemical parameters (see Figure 9) and serum levels of major proinflammatory cytokines (see Figure 10) demonstrated a postoperative reaction of the organisms on the second day after implantation. A marginal rise in fibrinogen, creatinine, and ALT on the second day after surgery may be indicative of a nonspecific bodily reaction to the surgical intervention or a reaction to the release of silver from the surface of the samples.

On the second day of the postoperative period, a 2.8-fold and 2.4-fold increase in TNF-α and IL-6 levels, respectively, were also observed (Figure 10). The reversion of the studied indicators to normal values by the seventh day of observation, and their subsequent maintenance within these limits at later stages, signifies the absence of a systemic reaction of the body to the implantation of samples.

The absence of any significant deviations of the studied indicators (Figure 8, Figure 9 and Figure 10) from preoperative values should be interpreted as the absence of a systemic reaction of the body to heterostructures with bimetallic nanoalloys, which indicates the high biocompatibility of the materials. The aim of this section of the study was not to demonstrate statistical differences between the groups, but rather to illustrate the rapid recovery process, whereby all key clinical indicators returned to physiological norms within the first week after surgery and remained within these limits throughout the entire three-month observation period.

In general, the selection of implantation times of 1 and 3 months constitutes a conventional and informative approach in preclinical biocompatibility studies, thereby enabling the observation of pivotal phases of the tissue response in accordance with ISO 10993-6 guidelines [48,49]. The 1-month period allows for the assessment of the acute and subacute phases of the inflammatory response to the implant. The 3-month period is pivotal for evaluating the transition to the chronic phase and the initiation of tissue response stabilization [50].

As demonstrated in Table 1, the results of the morphohistological analysis indicate that, following a period of three months, all samples exhibited a low level of rejection by the recipient’s body. The samples were characterized by the formation of a fibrous capsule without indurations, and the absence of signs of an active inflammatory reaction.

**Figure 11:**
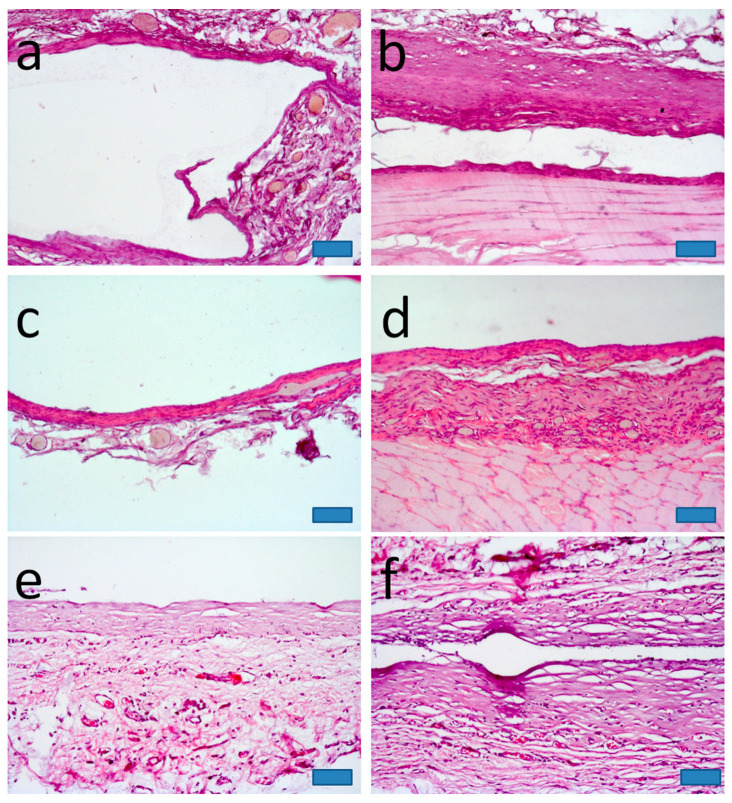
Microscopy images of fibrous capsule structures around implants after 1 month (AuAg/Ir/Ti ((**a**): capsule wall at the periphery; (**b**): the wall of the capsule adjacent to the muscle); AuAg/Au/Ti ((**c**): capsule wall at the periphery; (**d**): the wall of the capsule adjacent to the muscle)) and 3 months: AuAg/Ir/Ti (**e**), AuAg/Au/Ti (**f**) of subcutaneous implantation in rats (blue line is scale bare: 100 μm).

In the case of heterostructures with AuAg nanocomposites (AuAg/Ir/Ti and AuAg/Au/Ti), an uneven distribution across the thickness of the fibrous capsule walls was observed after one month (see Figure 11a–d). The capsule on the skin side (at the periphery) was found to be thinned with small clusters of capillary-type blood vessels (see Figure 11a,c), and in the deep layers, it was found to be densely fused with skeletal muscle, which exhibited varying densities of collagen fibers and more pronounced vascularization, also in the form of blood-filled capillaries (see Figure 11b,d). Three months after the implantation of the AuAg/Ir/Ti and AuAg/Ir/Ti samples, the capsule wall was uniform in thickness, with moderate collagen fiber density around the entire perimeter (Figure 11e,f). In certain regions, there was an abundance of vascularization, manifesting as clusters of capillaries of varying dimensions. Diffusely located lymphocytes were observed on the surface of the capsule and in its thickness.

Conversely, in the AgAu/Ir/Ti heterostructure, an augmentation of the implantation period to 3 months gave rise to a thickening of the packing density, accompanied by the formation of dense hyaline-like fields comprising a substantial number of fibroblasts and diffuse lympho-macrophage infiltration (see Figure S4).

A comprehensive histological assessment revealed that the nanoalloy on the Ir surface of the AuAg/Ir/Ti sublayer yielded the most favorable outcome among all samples. The relative effect was reproducibly observed both after one month and three months of implantation. The samples containing AuAg nanoalloys demonstrated the lowest degree of sclerosis and the highest degree of vascularization, indicating a satisfactory level of biocompatibility.

A thorough analysis of the composition of the extracted samples revealed that under in vivo conditions, both silver and gold undergo a gradual dissolution process, thereby ensuring the sustained release dynamics of the two active components. The proportion of dissolved silver is found to be approximately 22–30% and 36–40% after one and three months, respectively, and that of gold is found to be approximately 7–14% and 20–25%, respectively (see Table 2).

**Figure 12:**
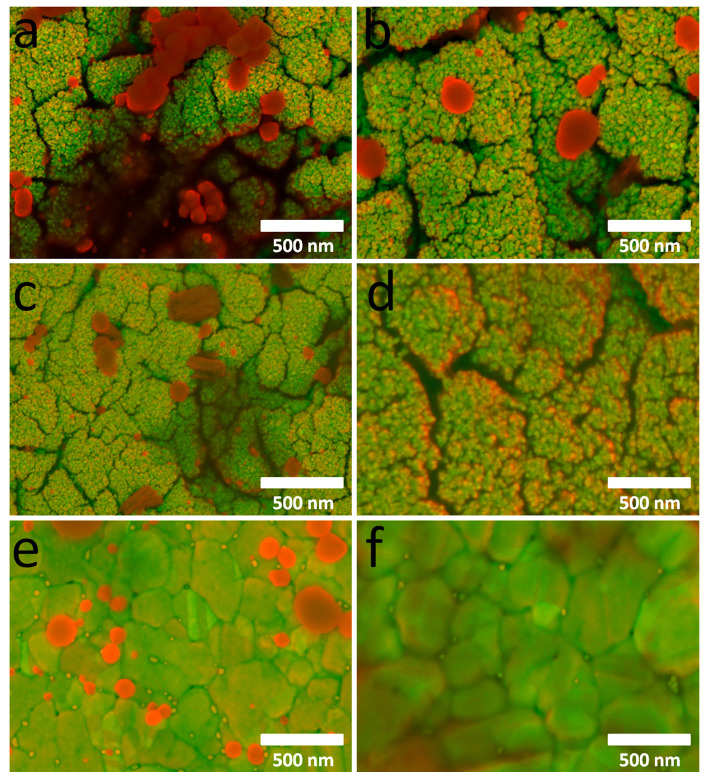
The SEM images of surface samples after 1 month and 3 months of subcutaneous implantation (Z contrast filter was used to assign the Ag component with red color fields, and Au, Ir components with green color fields): AgAu/Ir/Ti (**a**,**b**), AuAg/Ir/Ti (**c**,**d**), AuAg/Au/Ti (**e**,**f**).

Typical SEM images of the sample surfaces after implantation are shown in Figure 12a–f. After one month of implantation, large Ag agglomerates are observed on the surfaces of AgAu/Ir/Ti, AuAg/Ir/Ti, and AuAg/Au/Ti—red areas (Figure 12a,c,e). After 3 months of implantation, a decrease in these structural elements is observed on the surfaces of AuAg/Ir/Ti and AuAg/Au/Ti (Figure 12d,f), indicating their active dissolution during implantation. In the case of the “inverted” analog of AgAu/Ir/Ti, where an oxide phase is present in addition to the AgAu nanoalloy, rounded particles (red areas) are observed in the SEM image (Figure 12b), which are probably poorly soluble silver oxide. After 3 months of implantation, silver nanofilms and individual gold particles (green areas) are present on the surface of the samples (Figure 12b,d,f).

## 3. Discussion

In the majority of studies, solution [33,36] and green chemistry [27,28] methods are employed to obtain bimetallic silver–gold nanoalloys that are stabilized in suspensions or colloidal solutions. These suspensions and solutions are typically employed for the impregnation of implants [51], yet concerns regarding the uniformity of bimetallic coatings or nanoparticles’ distribution and their purification from the carrier matrix persist. The findings of this study demonstrated the feasibility of employing two step gas-phase methods (MOCVD and PVD) to synthesize AgAu and AuAg nanoalloys. It was first discovered that the order of metal deposition (AgAu or AuAg) significantly influenced the phase composition of film heterostructures of AgAu/Ir/Ti and AuAg/Ir/Ti, as well as their biological characteristics. The utilization of gas-phase processes engenders the potential for the application of these materials for the surface modification of 3D medical objects, including mesh implants. It is noteworthy that these processes have been shown to produce bimetallic nanoparticles and the film heterostructures AgAu/Ir, AuAg/Ir, and AuAg/Au at relatively low temperatures (200–250 °C).

A thorough analysis of the antibacterial characteristics was conducted, which revealed that samples with heterostructures AgAu/Ir, AuAg/Ir, and AuAg/Au ex-hibited bactericidal activity against both Gram-positive and Gram-negative bacteria. Given that gold is not released into the solution within the first 48 h under in vitro conditions, it can be concluded that the antibacterial activity of all the samples studied is due to the release of silver ions and the subsequent accumulation of their minimum concentration necessary to suppress bacterial growth. Notably that the growth of *P. aeruginosa* and *S. epidermalis* colonies was inhibited at 8 and 24 h, respectively. This suggests that higher concentrations of Ag^+^ ions are required to inhibit the growth of Gram-positive bacteria than for Gram-negative bacteria. These observations are consistent with literary data [52,53].

In general, the idea of noble metal-contained film heterostructures as new type of antibacterial materials is based on the galvanocouples between Ag and more noble metals (Ir and Au). In fact, the silver becomes a sacrificial anode and actively dissolves [21,54,55]. Thus, the dynamic of silver release depends on the cathode material and potential as well as silver chemical form, surface area, etc. Thus, herein we have shown that even partial blocking of the Ir surface by gold nanoparticles affects the dynamics of silver release, which was lower for AgAu/Ir/Ti sample than for the AuAg/Ir/Ti one. It is also interesting to consider the issue of changing the dynamics of antibacterial action via the Ag/Au ratio in the bimetallic active component. Note that in the long-term dynamics, the release of silver should destroy the nanoalloys and promote the release of gold in the form of nanoparticles or ions. In turn, gold may also contribute to antibacterial action, especially against Gram-positive bacteria (*S. aureus*) [56,57,58,59].

Indeed, under in vivo conditions, the release of both silver and gold from the surface of all samples with nanoalloys has been observed. It is noteworthy that the metal ratios (Ag/Au 1.6–2.9), dissolved after one and three months of implantation, are close to the specified metal ratios in the initial heterostructure samples (Ag/Au 1.8–2.3). This observation provides indirect confirmation of the formation of nanoalloys on the surface of heterostructures. The presence of nanoalloys in the samples enables more precise control of the mechanism of metal release from the surface of implants in vivo. In contrast to the findings in previously studied samples with Ag/Ir/Ti and Ag/Au/Ti heterostructures [23], the morphohistological profiles of heterostructures with AuAg nanocomposites demonstrate the increase in microvessel indices following three months of implantation (Table 2). This increase is a crucial indicator of implant survival. Indicators of inflammation or allergic reactions of the body to the implant (macrophages, lymphocytes, and mast cells) remain at a low level for AuAg/M/Ti samples during the three-month implantation period (Table 2).

## 4. Materials and Methods

### 4.1. Materials and Samples Preparation

MOCVD precursors, namely complexes [Ir(cod)(acac)], [(CH_3_)_2_Au(thd)], and [Ag(cod)(hfac)]_2_ with purity no less than 98% (Supporting Information, Table S1) and Au powder (99.99%) were used as noble metal sources. Ti-based disk samples assigned as “Ti” (Ti_6_Al_4_V; thickness 2 mm, diameter 10 mm; Baoji Chenyuan Metal Materials Co., Ltd., Baoji, China) were utilized as model implant material. Other materials are standard solutions of gold (MSDS, 170216, Merck, Rahway, NJ, USA), silver (MSDS, 119797, Merck, Rahway, NJ, USA), 0.01 M phosphate–salt buffer (pH = 7.2, 0.137 M NaCl, and 0.0027 M KCl, Eco Servise Ltd., Saint Petersburg, Russia), hydrochloric and nitric acids (Eco Servise Ltd., Saint Petersburg, Russia), and pure Ar, O_2_, and H_2_ gaseous (Chistye Gases Ltd., Novosibirsk, Russia). Double-sided foam tape has a 10 N/cm (HXDSF-8, Beijing Huaxia Yongle Adhesive Tape Co., Ltd., Beijing, China) tensile strength that was used to estimate the binding force of heterostructures to Ti disks.

The homemade MOCVD installation (Novosibirsk, Russia) with vertical reactor with cold walls and homemade UVM.71 PVD installation (Novosibirsk, Russia) were used for the deposition of film heterostructures and AgAu (AuAg) nanoalloys. Using MOCVD procedures (see details in Supplementary Information), Ir or Au coatings with thicknesses ~0.8 μm (Figure S1a–d) were deposited on Ti disks, giving the samples Ir/Ti or Au/Ti, respectively. Ag nanoclusters were obtained by MOCVD from [Ag(cod)(hfac)]_2_ (load 60 mg) at source temperature 120 °C and deposition temperature 250 °C. The precursor vapor was transported to the substrate by Ar gas (flow is 9 L/h). H_2_ was used as a gas reagent (flow is 12 L/h). The total pressure was 5–5.7 Torr and experiment time was 90 min. Au nanoclusters were obtained by PVD from Au powder (load 1 mg) heated up to 1670 °C. The deposition process occurred at U = 500 V, I = 500 mA. The precursor vapor was evaporated to the deposition zone under vacuum 8 × 10^−7^ Torr, and Au nanoclusters formed on substrates heated to 200 °C.

### 4.2. Methods

The composition and morphology of all samples (Figure 1) were studied by the set of standard methods (Table 3).

### 4.3. Dynamic of Metal Ions Release

The dynamics of Ag^+^ and Au^+^ ions’ release from the surface of AgAu/Ir/Ti, AuAg/Ir/Ti and AuAg/Au/Ti heterostructures were studied using the following procedure. For each sample, six parallels (analogs obtained at the same experimental conditions) were prepared and placed in five different polypropylene test tubes containing 5 mL of buffer. The test tubes were tightly capped and kept for 2, 4, 8, 24, and 48 h. After the corresponding time point, the analytical signals of Ag^+^, Au^+^ ions were recorded using the ICP-OES method.

### 4.4. Biological Studies

#### 4.4.1. Antibacterial Activity

The antibacterial activity of samples toward Gram-positive (*S. epidermalis*) and Gram-negative (*P. aeruginosa*) bacterial colonies was studied. For this, the standard solutions containing 2 × 10^6^ CFU/mL each were prepared in RPMI-1640 medium (Thermo Fisher Scientific, Waltham, MA, USA) with the addition of 0.3 g/L L-glutamine and 2 g/L sodium bicarbonate (Thermo Fisher Scientific, Waltham, MA, USA). The bacterial suspension (50 μL) was applied as a drop to each test sample and incubated in a humid thermostat at 37 °C. The next day, a drop of bacterial suspension from the sample was transferred to a Petri dish with the appropriate culture medium and cultured further for 2, 4, 8 or 24 h to evaluate the bacterial growth. The Axioskop 40FL microscope (Carl Zeiss, Jena, Germany) was used to count surviving bacterial colonies. The antibacterial activity of samples was determined in each time point via counting the surviving bacterial colonies stained with the BactoView Live Red kit (Biotium, Fremont, CA, USA). Measurements in each experimental group were performed in three replicates.

#### 4.4.2. Biocompatibility In Vivo

In order to assess the biocompatibility of samples in vivo, subcutaneous implantation was used, followed by removal of materials at various observation times. Laboratory rats were used as test systems for experimental studies (see details in Supplementary Information).

The studies encompassed the examination of the cytological, biochemical, and cytokine composition of the animals’ peripheral blood at 2, 7, 14, 30, and 90 days after surgery. A total of 250 μL of blood was collected from the tail vein into tubes containing potassium EDTA, a coagulation activator, or 3.2% sodium citrate. The cytological composition of rat blood after implantation was examined using a BC-2800 Vet veterinary hematology analyzer (Mindray, Shenzhen, China) in accordance with the manufacturer’s recommendations. Biochemical studies (fibrinogen, creatinine, urea, and ALT) were performed using a Dry-Chem 4000i biochemical analyzer (Fuji, Tokyo, Japan) in accordance with the manufacturer’s recommendations. The cytokine composition of the blood was then assessed for the presence of the inflammatory markers TNF-α, IL-1β, and IL-6, using the corresponding ELISA reagent kits (Vector-Best, Novosibirsk, Russia) according to the manufacturer’s instructions.

#### 4.4.3. Histological Studies

The collection of biological materials was executed at one- and three-month intervals. Samples were extracted in conjunction with the surrounding connective tissue capsule and fixed with a 20-volume excess of 10% buffered formalin (Biovitrum, St. Petersburg, Russia) for a period of 48 h. The implants were then meticulously extracted, and the fibrous capsule material was subjected to histological processing and embedded in paraffin blocks using the standard method. From the paraffin blocks, 5 μm thick sections were prepared on an HM340E rotary microtome (Microm, Walldorf, Germany), following the staining procedure recommended by the manufacturer of the hematoxylin and eosin (Biovitrum reagent kits, Russia), using Biomount mounting medium (Biovitrum, Russia). The histological analysis was performed using an Axioskop 40FL microscope (Carl Zeiss, Oberkochen, Germany) equipped with an ADF Pro 08 color camera and ADF ImageCapture software v.1.0. (ADF Optics, Shenzhen China). The scale system (0–3 points; see Supporting Information) was utilized to estimate morphohistological changes during histological experiments.

#### 4.4.4. Statistical Analysis

The quantitative data were processed using Statistica 13.0 (TIBCO Software Inc., Palo Alto, CA, USA). The normality of the datasets in each experiment was tested using the Shapiro–Wilk test. Student’s *t*-test was applied to identify disparities between groups. The statistical significance was set as *p* < 0.05. The results presented in the graphs are expressed as the mean value ± standard deviation.

## 5. Conclusions

The surface modification of Ti-based implants with heterostructures consisting of an M (Ir or Au) sublayer and bimetallic nanoalloys (AgAu or AuAg with Ag/Au ratios of 1.8–2.3) was achieved using gas-phase deposition methods. The bimetallic solid solutions are formed if the gold is secondly deposited component (AuAg/Ir/Ti and AuAg/Au/Ti samples) while a mixture of AgAu and Ag_2_O phases are observed if the silver is secondly deposited component (AgAu/Ir/Ti samples). The proportion of Ag^+^ ions released from the surface after 2–48 h was found to be greatest for the Ir sublayer (AuAg/Ir/Ti sample) and smallest for the Au sublayer (AuAg/Au/Ti sample), with no release of Au^+^ ions recorded during this time interval. All heterostructures inhibited the growth of *P. aeruginosa* after 8 h, while the growth of *S. epidermidis* on the sample surfaces was inhibited after 24 h in most cases. Notably that the AuAg/Ir heterostructures showed the most pronounced total antibacterial effect within the 8 h.

Under in vivo conditions, silver and gold ions were released from the surface of all samples. The metal ratios (Ag/Au 1.6–2.9) released after one and three months of implantation were close to the initial sample ratios. Despite the release of Au^+^ and Ag^+^ ions, no systemic reactions to the heterostructures containing bimetallic nanoalloys were detected, indicating the high biocompatibility of the materials. Three months after implantation, an increase in the number of microvessels was observed in the morphohistological profiles for the AuAg/M/Ti sample, while the indicators of inflammation or allergic reactions to the implant remained low, confirming harmonious integration within the host tissue.

Thus, AuAg/M film heterostructures hold promise for providing improved biological properties to implants. In further studies, varying the silver-to-gold ratio may fine-tune the desired biological properties.

## Figures and Tables

**Table 1:** Summary of findings during histological examination of materials.

Samples	Packing Density		Microvessels		Macrophages		Lymphocytes		Mast Cells	
	1 m	3 m	1 m	3 m	1 m	3 m	1 m	3 m	1 m	3 m
AgAu/Ir/Ti	1	3	1–2	0	1	2	1	2	2	0
AuAg/Ir/Ti	3	2	1	2–3	1	1	1	2	1	1
AuAg/Au/Ti	1–3	1	0–2	3	1	1	1	1	1	1

**Table 2:** Silver and gold content (μg/cm^2^, ICP-OES) in implants with heterostructure coatings after 1 and 3 months of implantation.

Samples	Metal (Implantation Period)					
	Ag (Intial)	Au (Intial)	Ag (1 m)	Au (1 m)	Ag (3 m)	Au (3 m)
AuAg/Ir/Ti	9.7	5	6.7	4.3	5.8	4.0
AuAg/Au/Ti	12	5.5	9.4	5.1	7.6	4.4
AgAu/Ir/Ti	11	5.8	7.7	5.3	6.7	4.2

**Table 3:** The samples’ characterization: methods, equipment and experimental details.

Method	Equipment and Experimental Details	Interpretation
Inductively coupled plasma atomic emission spectroscopy (ICP-OES)	A high-resolution spectrometer iCAP 6500 (Thermo Fisher Scientific, Waltham, MA USA) was used. The registration of samples was performed at the axial observation of plasma: cooling argon flow was 12 L/min, secondary was 0.5 L/min, nebulization was 0.7 L/min, registration time was 5 s, and power supplied to an ICP inductor was 1150 W.	The as-deposited samples (Figure 1) and samples after implantation were dissolved in HNO_3_ and HCl acids, and then analyzed. The Ag and Au content in the samples were calculated using the most intense analytical lines: 242.795, 267.595 nm for Au and 328.068, 338.289 nm for Ag.
X-ray photoelectron spectroscopy (XPS)	FlexPS spectrometer (SPECS, Berlin, Germany), PHOIBOS-150 analyzer (SPECS, Berlin, Germany) with 1D-DLD detector (SPECS, Berlin, Germany), FOCUS-500 monochromator (SPECS, Berlin, Germany), Al Kα radiation, hv = 1486.71 eV, 14 kV, 200 W. The calibration of binding energies conducted via the Fermi level of the valence band (0.0 eV).	CASA program version 2.3 software (Tokyo, Japan) was used. The Au 4f and Ag 3d spectra deconvolution was performed using the Functional Lorentzian (LF) lineshapes. For Ir peak fitting, the fine-tuning parameters were applied according to [60]. Other spectra were fitted by the Gaussian–Lorentzian product functions. Subtracting the background was utilized via the Shirley method.
Scanning Electron Microscopy (SEM)	HITACHI UHR FE-SEM SU8200, Hitachi, Ltd., Hitachi, Tokyo, Japan (3 keV, LA detector)	To determine Ag, Au clusters sizes, SEM images of the samples (Tiff files) were imported into the program image analysis version 1.54 software (ImageJ) National Institutes of Health, 9000 Rockville Pike, Bethesda, Rockville, MA, USA (version 2.0.0)
Scanning probe microscope with surface topology registration in atomic force microscopy mode (AFM)	CMM-2000 microscope (№46918), Manufacturer: Ltd. PROTON, Zelenograd, Russia (https://www.z-proton.ru (accessed on 8 December 2025))	The probes used were CSG-01 cantilevers(NT-MDT Tips, Moscow, Russia) with a tip curvature radius of 10 nm. The achievable resolution was up to 4 nm for lateral relief dimensions and up to 0.02 nm for relief heights. Sample roughness parameters were calculated using the CMM-2000 microscope software (Proton-MIET Plant Ltd., Moscow, Russia).

## Data Availability

The original contributions presented in this study are included in the article/Supplementary Materials. Further inquiries can be directed to the corresponding author.

## Supplementary Materials

The following supporting information can be downloaded at: https://www.mdpi.com/article/10.3390/ijms262411945/s1. The references [61,62,63] are given in supplementary files.
