# Supplementary material for: Combination of Noble Metal and Gold–Silver Nanoclusters as Enhanced Antibacterial Coatings for Ti-Based Medical Implants
Source: Int J Mol Sci. 2025 Dec 11;26(24):11945. doi: 10.3390/ijms262411945 (PMC12732423; doi:10.3390/ijms262411945)
Supplement: Supplementary file 1 [file ijms-26-11945-s001.zip › ijms-3989893-supplementary.pdf]

Supporting information to

Combination of noble metal and gold-silver nanoclusters as enhanced antibacterial coatings for Ti-based medical implants

Evgeniia S. Vikulova [1,*], Svetlana I. Dorovskikh [1], David S. Sergeevichev [2], Tatiana Ya. Guselnikova [1], Anastasiya D. Fedorenko [1], Alexander A. Zheravin [2] and Natalya B. Morozova [1,2]

1. Nikolaev Institute of Inorganic Chemistry Siberian Branch of the Russian Academy of Sciences, 3 Lavrentiev Ave., 630090 Novosibirsk, Russia; lazorevka@mail.ru (ESV), reter16@yandex.ru (SID), tguselnikova@niic.nsc.ru (TYaG), fedorenko@niic.nsc.ru (ADF), mor@niic.nsc.ru (NBM);

2. «E. Meshalkin National Medical Research Center» of the Ministry of Health of the Russian Federation, 15 Rechkunovskaya Str., 630055 Novosibirsk, Russia; d_sergeevichev@meshalkin.ru (DSS), zheravin@meshalkin.ru (AAZ)

- Correspondence: lazorevka@mail.ru

Experimental Section

Table S1. Characterization of the reagents used for heterostructure preparation

Chemical name	Source	Purification	Purity
Acetylacetonato(1,5-cyclooctadiene)iridium(I) **[Ir(cod)(acac)]** CAS 12154-84-6	Synthesized by us according to procedure [a]	Double sublimation (1.33 Pa, 110°C)	>98%
2,2,6,6-tetrametylhexane-3,5-dionato(dimethyl)gold(III) **[$(CH_3)_2$Au(thd)]**	Synthesized by us according to procedure [b]	Recrystallization from hexane	>98%
Dimer (1,5-Cyclooctadiene) (hexafluoroacetylacetonato)silver(I) **[Ag(cod)(hfac)]$_2$** CAS 38892-25-0	Synthesized by us according to procedure[c]	Recrystallization from tolulene	>98%

[a] Vikulova, E. S., Ilyin, I. Yu., Karakovskaya, K. I., Piryazev, D. A., Turgambaeva, A. E., Morozova, N. B. (**2016**). Volatile iridium(I) complexes with β-diketones and cyclooctadiene: syntheses, structures and thermal properties. *Journal of Coordination Chemistry*, 69(15), 2281–2290. https://doi.org/10.1080/00958972.2016.1198955

[b] Parkhomenko, R. G., Morozova, N. B., Zharkova, G. I., Shubin, Y. V., Trubin, S. V., Kriventsov, V. V., Igumenov, I. K. (**2012**). Deposition of Au thin films and nanoparticles by MOCVD. *Chemical Vapor Deposition*, 18(10-12), 336- 342.https://doi.org/10.1080/00958972.2016.1198955

[c] Vikulova, E. S., Il'in, I. Y., Sukhikh, T. S., Artamonova, P. K., Morozova, N. B. (**2023**). Complexes of Silver 1, 1, 1, 5, 5, 6, 6, 6-Octafluorohexane-2, 4-dionate with π-Donor Ligands: Synthesis, Structure, and Thermal Properties. *Russian Journal of Coordination Chemistry*, *49*(11), 743-752.

The details of MOCVD of Ir and Au films were described in [*doi:10.3390/ijms25021100*]. In brief, porous Ir coatings with thicknesses 1 μm were obtained on Ti discs from [Ir(cod)(acac)] at deposition temperature 310°C, evaporator temperature 110°C, Ar and O_2 flow rates 2 l/h, total pressure 1 Torr, precursor load 60 mg, deposition time 120 min (Fig S1a-b). Porous Au coatings with thicknesses 0.8 μm were obtained on Ti discs from $(CH_3)_2Au(thd)$ at deposition temperature 270 °C, evaporator temperature 60°C, Ar and O_2 flow rates 2 l/h, total pressure 1 Torr, precursor load 80 mg, deposition time 120 min (Fig S1c-d).

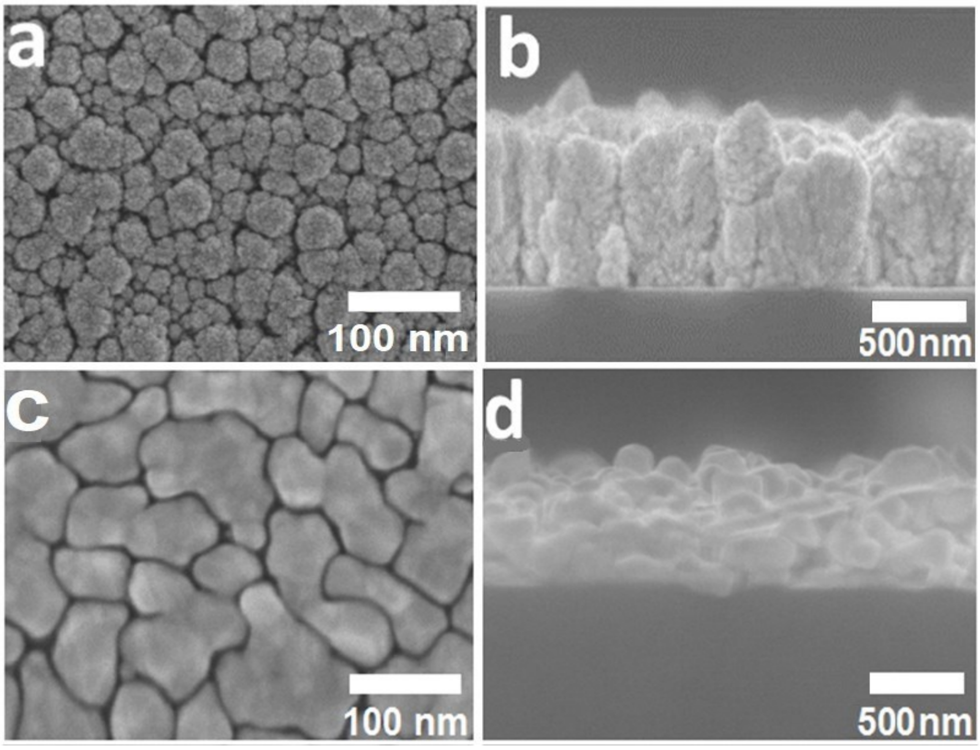

Figure S1. Surface and Cross-section SEM images of Ir coatings (a,b) and Au coatings (c,d)

The in vivo studies of biocompatibility were approved by the local ethics committee of the E. Meshalkin National Medical Research Center of the Ministry of Health of the Russian Federation. The study protocol was conducted in accordance with the recommendations for the proper use and care of laboratory animals (European Communities Council Directive 86/609/EEC and the principles of the Declaration of Helsinki). Wistar rats (weight 130–150 g, n = 8) were used for experiments.

All experimental studies on animals were conducted in an operating theatre under anaesthesia, in compliance with aseptic and antiseptic procedures. Twelve hours before the planned intervention, the animal was stopped feeding and transferred to a water-only diet. A veterinarian examined the animal and confirmed that it was in satisfactory condition. General anesthesia was induced via the intramuscular administration of Zoletil-100 (Virbac, France) at a dose of 50 mg/kg. Next, the hair on the back was shaved off (an area measuring approximately 5 x 5 cm) and the animal was transferred to and

secured in a prone position on the operating table. The surgical site was cleaned with an antiseptic solution and a longitudinal skin incision measuring up to 1 x 1 cm was made to the side of the spine. A pocket was formed in the subcutaneous tissue using surgical scissors in a "blunt manner" to place the sample under investigation. The skin incision was sutured with absorbable thread and treated with an antiseptic solution. The animals were transferred to cages and observed in the vivarium for either 30 or 90 days, after which they were euthanised.

Results Section

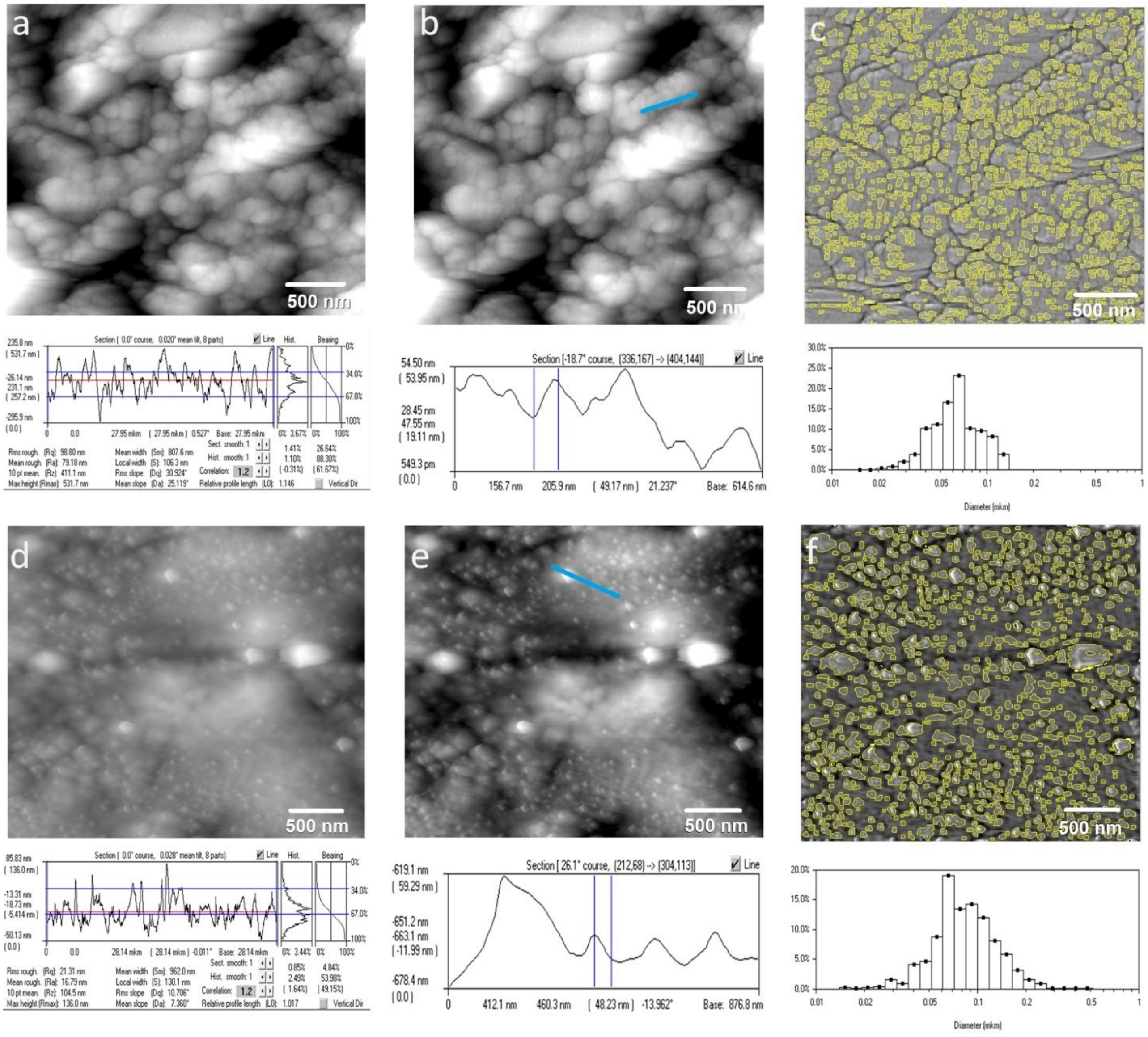

Figure S2. AFM images of surface of AgAu/Ir/Ti with a roughness profile (a), with the grain section profile (b), with differential grain diameter distribution profile (c), and AFM images of surface of Ag/Ir/Ti with a roughness profile (d); with the grain section profile (e), with differential grain diameter distribution profile (f)

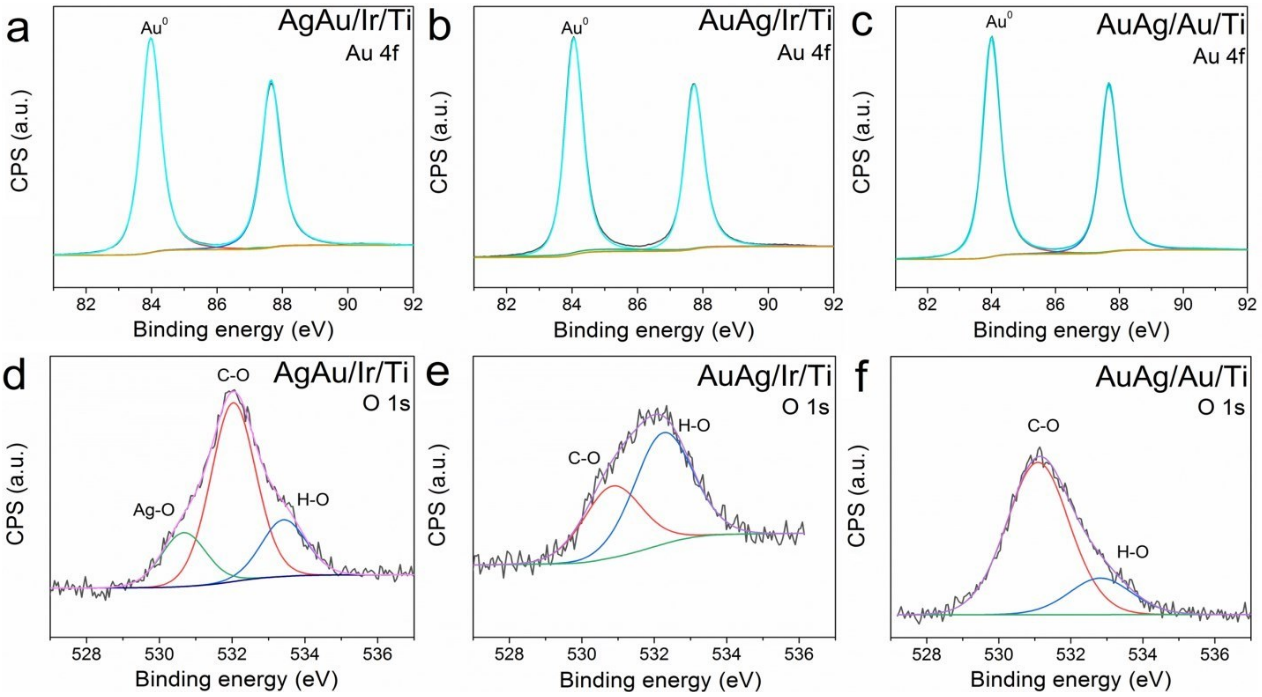

Figure S3. XPS Au 4f spectra of samples AgAu/Ir/Ti (a), AuAg/Ir/Ti (b), AuAg/Au/Ti (c) and O 1s spectra of AgAu/Ir/Ti (d), AuAg/Ir/Ti (e), AuAg/Au/Ti (f)

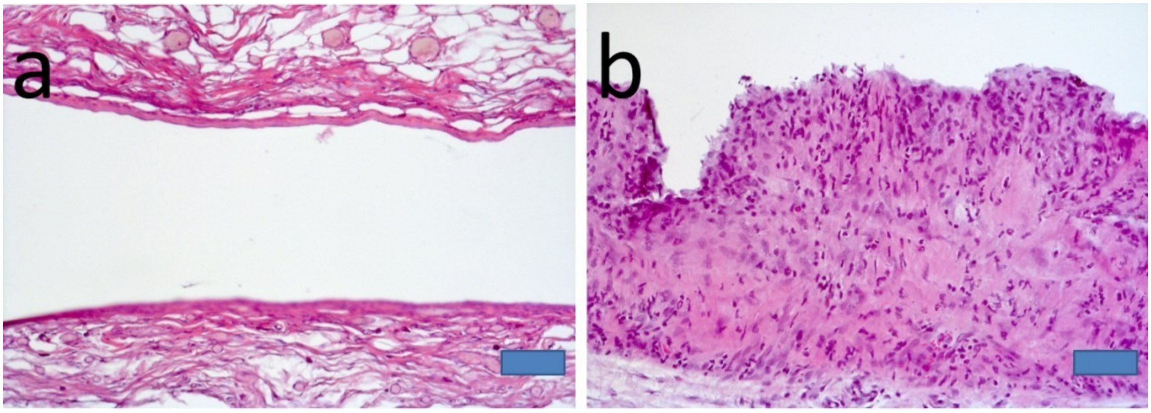

Figure S4. The microscopy images of capsule wall structures of implant AgAu/Ir/Ti sample after 1 (a) and 3 months (b), bare is 5 μm

Table S2. Average number of bacteria colonies counts after incubation at different times

Sample	*S. epidermalis*				*P. aeruginosa*			
	**Incubation time, hours**							
	**2**	**4**	**8**	**24**	**2**	**4**	**8**	**24**
AgAu/Ir/Ti	980.3±28.9	844.7±58.5	479.7±69.2	0	989.0±28.6	831.0±58.6	0	0
AuAg/Ir/Ti	947.3±29.0	832.7±57.6	12.7±8.5	0	958.3±59.8	847.7±52.4	0	0
AuAg/Au/Ti	956.7±41.8	802.7±47.0	308.0±21.3	0	888.3±27.0	818.3±55.5	0	0
